# 

**Published:** 1887-09-03

**Authors:** 


### CONTENTS.

**The New Journalism** - - - - - - 373

**Words of Consolation.**—Chapter XLVIII. The Sufferings of Christ - - - - - 374

**The Doctor's Pulpit.**—Scarlet Fever - - - - 375

**Visiting Day.** By R. G. Emery - - - - - 377

**Lunatic Literature.** By a Voluntary Commissioner - - - - - - 378

**Notes and News.**—Miscellaneous Items.—Scarlet Fever in the Metropolis.—Hospital Sunday Church Collections at Lowestoft.—"Queen Victoria's Bounty."—Church Parade Collections.—Vacancies.—The Tavistock Cottage Hospital.—Honorary Hospital Appointments.—The Gloucester Free Hospital, etc., etc. - - - - 380

**Annotations.**—The Queen's Fund and the Pension Fund.—Twenty Thousand Hospital Supporters.—Working Men and their Manners - - - - - 383

**Kate Mountstephen.** By C. G. Furley.—Chapter II. "An Ounce of Practice" - - - - 384

**A New Home Hospital.** By Shirley F. Murphy, M.R.C.S. - - - - - - - 387

**Native Medical Practice in India.** By a Major-General of the Bengal Army - - - - - 387

**Questions and Answers** - - - - - 388

**The National Pension Fund** - - - - 388

**The Editor's Letter-Box.**—The Norfolk and Norwich Hospital.—A "Matron's Corner."—The Mission Parcel Society - - - - - - - 389

**Till the Doctor Comes.**—XXV. Something about Eyes.—Squint.—Spectacles.—General Remarks - - 389

**The Children's Corner.**—Puzzles, Answers, Letters, etc. - - - - - - - 390

**Amusements with Profit** - - - - - 390

